# Digital-Media-Based Interaction and Dissemination of Traditional Culture Integrating Using Social Media Data Analytics

**DOI:** 10.1155/2022/5846451

**Published:** 2022-05-31

**Authors:** Na Hong

**Affiliations:** ^1^School of Literature, Shandong University, Jinan, Shandong 250100, China; ^2^School of Foreign Languages and Literature, Shandong University, Jinan, Shandong 250100, China

## Abstract

This paper adopts the approach of the Internet fusion digital media to conduct in-depth research and analysis on the interaction and communication of traditional culture. This structural information can be used to express and describe the Internet requirements. With the increasing demand for the Internet applications, the number of objects in the network and the complexity of the structure are also growing, so we can design suitable clustering algorithms to obtain more reasonable network structure information by using different semantic information in the heterogeneous information network. Firstly, the caching scheme is transformed into a backpack problem, and the storage scheme is obtained recursively by using dynamic programming, after which the computational task is optimized based on this storage scheme by using the whale algorithm to make it the least costly. The exact balance between the connotation essence of traditional culture and the emotional resonance in modern life has not yet been found, and the visual language belonging to cultural aesthetics under the combination of traditional and modern aesthetics needs to be explored for a long time. From an objective point of view, the digital art communication of cultural elements still suffers from five problems: lack of communication cultivation and stratification of target audiences, low quality of transcription of digital artworks, unbalanced economic and cultural values, uneven development of digital art communication forms, and low degree of Internet adaptation. A ranking algorithm based on fusing multiple meta-paths is proposed to better reflect the ranking values of different object distributions of the Internet data in heterogeneous information networks, which can obtain better clustering, while better clustering can be calculated to obtain more reasonable rankings, and finally, the types of the Internet-oriented applications are divided into multiple reasonable clusters. The effects of selecting different meta-paths on the clustering algorithm and the effects of fused meta-paths versus single meta-paths on the clustering results are given in the experimental results section. Also compared with other clustering algorithms, the I-RankClus algorithm can better achieve the data clustering classification for the Internet application scenarios.

## 1. Introduction

In the rapid development of the Internet of Things, the number and interaction of sensors and smart devices in the network are increasing. Daily life is covered by a variety of complex and diverse the Internet data, and all kinds of the Internet data have their unique properties, and the Internet data within a certain range form a complex system that intermingles and connects [1]. In the face of such an intricate system, it is often difficult for users to find the required resources quickly and accurately, so it is necessary to study efficient and intelligent methods to mine the information among the Internet data, and data mining provides researchers with the corresponding methods. The higher stage of the Internet era has brought greater opportunities and created the best conditions for digital art communication of traditional culture [2]. The deepening popularity of the Internet, especially the rise of the mobile Internet, has brought us to a higher stage of the Internet era. While it is transforming communication relationships and reshaping social forms, it is also bringing tremendous opportunities for the development of digital art communication of cultural elements. The Internet data can be divided into static data and dynamic data; static data is mostly label type and address type data; RFID generated data is mostly static data; static data is mostly stored in a structured, relational database; dynamic data is a time sequence of data; dynamic data of the Internet is characterized by each data having a one-to-one correspondence with time, and this relationship is particularly important in data processing; this type of data storage is usually stored in a time-series database way. The rapid progress of information technology, the penetration of the Internet into people's lives, the spread of new applications and mobile smart terminals, and the emergence of new technologies such as cloud computing have all brought people into the Internet era in the true sense of the word. At the same time, people's lifestyles and cognitive patterns are also undergoing huge changes under the influence of Internet [3]. Various intelligent terminal devices have become the necessities of people's lives, the Internet has become the most important channel for knowledge and information dissemination, and the in-depth development of mobile Internet and smartphones, their powerful functions, and the full use of fragmented time has made people rely on cell phones to an unprecedented extent.

With the rapid development of multimedia art fusion of technology and art, as well as the huge increase in information production capacity of data storage technology combined with computer and Internet, resulting in a dramatic increase in the number of information resources of culture and art, users' thirst for cultural knowledge in-depth, real-time, integrated resources are increasingly in demand, and the functions and service mode of traditional cultural museums can no longer adapt to the development of the times. The data mining is generally the process of automatically searching a large amount of data for information with special relationships hidden within it. In most cases, data mining involves extracting data from a data warehouse into a data mining repository, or, of course, from other databases or flat files, except that data cleaning in a data warehouse is similar to data cleaning in data mining, in that if the data has already been cleaned when it is imported into the data warehouse, there is no need to clean the data again when performing data mining, and all data can be applied directly to the mining algorithm. The data can be directly applied to the mining algorithm. Research has shown that the interdependence of social media motivations and the impact of social media use among product-based and service-based SMEs is positive but unstable [4]. In addition, SMEs offering physical products are more likely to use social media based on cost-benefit motivations, while service-based SMEs are more likely to view interactivity as a key motivation. New media is an engine for business development and an indispensable technological tool for information interaction between companies and users, linking companies and users more conveniently and efficiently regardless of time and space constraints. The popularity of smartphones and tablet computers has facilitated the application of new media for enterprises to interact with users, and the development of new media technology and the increasingly rich variety of platforms have improved the effectiveness of information interaction between enterprises and users. Compared with traditional media, new media is closer to users, with the characteristics of mobility, interactivity, personalization, etc., which is the interaction method users prefer to use.

In this paper, the influence of each type of object in a heterogeneous information network is calculated based on meta-paths, and the calculated influence can reflect the network structure information contained in meta-paths [5]. There are multiple meta-paths in the heterogeneous information network structure, and a single meta-path cannot reflect the complete structural information of the heterogeneous information network. This paper fuses multiple meta-paths and calculates the influence of the fused meta-paths, and the calculation results can reflect the structural information of the heterogeneous information network. In the traditional exhibition hall, the amount of information disseminated by culture has certain limitations, and the way of the display is mostly two-dimensional flat display, the audience can only passively receive information through the display content, and the degree of information received cannot be controlled. The audience can only passively receive information through the display content, and the degree of information received cannot be controlled. Both the visual and interactive aspects have a poor experience. However, the exhibition hall under the perspective of artificial intelligence is to use new technology and new interactive means to show and create the atmosphere of the exhibition hall and create a new exhibition space. Through the application of intelligent technologies such as virtual and reality technology, image capture, and cloud intelligence, we adopt a visual design language that conforms to the form to create a modern pavilion that meets the audience's experience, enhance the sense of novel and flexible visual experience, and present it to the audience with a more intelligent visual design. The explosion of the mobile Internet has promoted a change in mass cultural communication, with a clear shift in marketing discourse and ideology to the masses, providing new content, carriers, and paths for urban marketing, while providing new development opportunities for more cities and vast rural areas in China, shaping a more comprehensive and three-dimensional image of the landscape.

## 2. Related Works

Under the perspective of cultural self-confidence, the value of cultural elements is affirmed, and it is believed that cultural elements refer to relevant elements from a traditional culture that differentiate from other countries and regions and highlight the national characteristics of culture, and are things, phenomena, and spirits that have been gradually accumulated in the long-term historical development process, with long-term continuity, era, and internationalization, and are a positive symbol and spiritual image [6]. It is also proposed that the inheritance and promotion of cultural elements have various motives: one is close to the current demands of people, the second is the market drivers of commercialization, and the third is the spiritual pull of revival. It also summarizes and extends that the basic characteristics of cultural elements also include that they are truths and excellent values of universal significance and are the result of long-term national self-awareness and self-screening [7]. This article echoes the needs of modern society in the context of cultural self-confidence, and thus the concept of cultural elements is of certain practical significance, but it does not propose effective strategies for the dissemination and development of cultural elements, and it can be seen that the research on the application of cultural elements is still mostly focused on the cultural perspectives of different industries, and there is still less research on the systematic digital dissemination of specialized cultural elements [8]. In general, although there are certain achievements and progress in the definition of the concept of cultural elements and related research, in the face of the rapid development of the Internet in recent years and the social shaping and communication changes brought about by it, the concept and development of cultural elements are bound to produce certain developments, especially for the significance of the international communication of cultural elements and the construction of the national image, which are bound to be extremely important, but the related research on cultural elements [9]. The market and management innovations based on the Internet platform, which brings important opportunities for the innovative development of traditional industries and also promotes the innovative development of the media industry, should adopt corresponding innovative strategies in terms of the innovation of production mechanism, marketing strategy, service, and experience of media products. The media industry promoted by “Internet+” presents the development trend of normalization of new technology application, diversification of business model, and standardization of policy management, which provides inspiration and reference for the operation and management of China's media industry. However, it is clear that the research on cultural elements has not developed accordingly with the progress of the times and the needs of communication, and this study is intended to fill the relevant gaps [10].

Digital media art is an interdisciplinary and integrated discipline that combines the natural sciences, social sciences, and humanities, embodying the concept of “science, art, and humanities.” The term digital technology reflects its technological basis, media emphasizes its foothold in the media industry, and art specifies all areas of application for the creation of artworks designed by it and the artistic design of digital media products [11]. It is argued that cultural heritage institutions (such as fine art galleries) should continuously increase their use of digital media to achieve a more effective presentation of their exhibits. The editors have effectively expanded the functional interfaces of the exhibition halls, making the design of the halls more fluid and robust based on multi-touch technology, and the exhibition formats can thus match and support more forms of application terminals and data formats [12]. Many foreign digital exhibition halls have accurately positioned their exhibition formats, and millions of halls have been promoted on the web, where intelligent media-based technologies are gradually emerging.

Digital media art has its unique artistic characteristics. In addition to the basic attributes of traditional art, its uniqueness is mainly expressed in “technology-based art,” such as immersive art, virtual reality art, network art, and other art forms closely related to technology. With the continuous development of digital technology, there will be more changes in digital media art [13]. As more and more cultural content becomes accessible resources, the “online” access to culture has given the right to choose culture to content platforms. Under the underlying logic of intelligent algorithmic technology, individuals who lack media literacy are at risk of being imprisoned in an “information cocoon,” repeatedly consuming homogeneous cultural content. Those who are relatively culturally disadvantaged are likely to develop polarised cultural tastes and even become cultural monopoles, which in the long run will affect the uplifting of the spirit of the times, the improvement of moral standards, and the enhancement of cultural literacy in society as a whole. With the application of digital pavilions and the accumulation of practical experience in research, digital pavilions have developed from a single concept at the beginning toward a systematic and comprehensive theoretical system and have accumulated valuable experience repeatedly in the process of practice. Nevertheless, due to the imperfect development of technology, there are still problems of interactivity and flexibility in the actual application cases of digital pavilions. With the rapid promotion of the artificial intelligence era, big data technology, optical imaging technology, image augmentation, and virtual reality technology are also widely used in the visual design of the exhibition hall, which has also changed the interactive approach and way of thinking of the exhibition design to a certain extent, from the boring single visual design form gradually to the comprehensive design form of technology and art integration. Social media refers to websites and technologies that allow people to write, share, evaluate, discuss, and communicate with each other and are tools and platforms for sharing opinions, insights, experiences, and perspectives with each other. The two main components of social media are spontaneous communication and large numbers of people.

## 3. Analysis of Interactive Communication of Traditional Culture in Digital Media Fused with the Internet of Things

### 3.1. The Internet Convergence Digital Media Technology Design

Due to the diversity of meta-paths in heterogeneous information networks, selecting only a single meta-path for calculation will lose the structural information of the object on other meta-paths, resulting in a lack of information integrity [14]. To avoid this situation, this paper uses a linear weighting method to fuse multiple single meta-paths for influence calculation. The influence calculation formula for fusing multiple meta-paths is shown as follows:

(1) $$\begin{array}{c} S\text{Ranks}\left(S_{i}\right)=\sum_{p_{i}\in P}w_{p_{i}}^{2}*S_{p_{i}}\text{Rank}\left(S_{i}\right), \end{array}$$

where *p*_*i*_ represents the selected single meta-path, *w*_*p*_*i*__^2^ is the path weight and *S*_*p*_*i*__Rank(*S*_*i*_) represents the influence calculated in the selected single meta-path *p*_*i*_.

When calculating the influence of an attribute type object *S*, a single meta-path SRS, SCS can be selected for weighted fusion SRS + SCs, which can retain both user and third-party influence on the influence calculation. The selection of different path weights *w*_*p*_ will also affect the final influence calculation results. The size of the path weights represents the importance of the current meta-path for the influence calculation, and the sum of the path weights is 1; i.e.,

(2) $$\begin{array}{c} w_{p_{1}}-w_{p_{2}}-w_{p_{3}},\ldots ,-w_{p_{i}}=1. \end{array}$$

**Figure 1:**
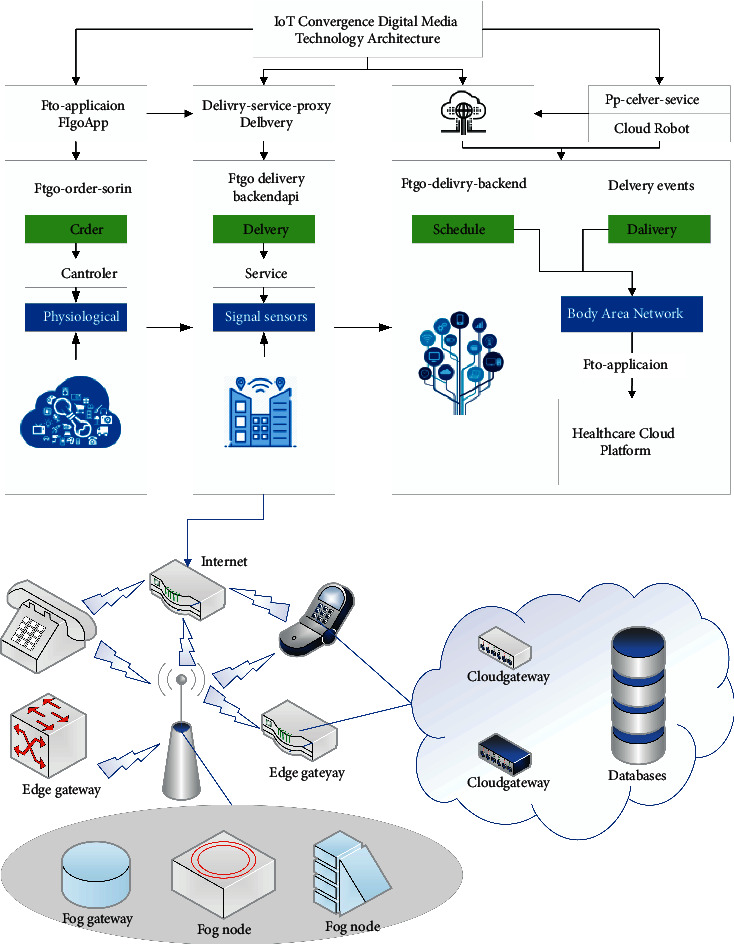
The Internet convergence digital media technology architecture.

One of the main tasks of digital culture is to study the operation mode of parallel reciprocity of digital cultural resources public service as well as a commercial operation, cultural resources, digital resources, key technology research, system integration solutions, and digital resource management and service platforms of various types of cultural institutions. In this “one museum system” virtual exhibition hall, the cost of services is effectively controlled, various effective resources are utilized, the audience is effectively expanded, and the government's cultural benefit policy is widely realized. Letting digital culture enter thousands of households will eventually become a reality. In today's world where communication power is productivity and creativity, it is to establish an international communication system that is balanced and matched with its political status, economic strength, and international influence to be able to fully present cultural civilization. Through the application demonstration role, we establish scalable, distributed, and interoperable digital cultural resource management as well as service platforms with various types of cultural institutions and carry out full-media digital resource services. At present, the digitization of our cultural museums is in order, but the depth and breadth of construction are still insufficient. Since public cultural centers can only provide on-site services during working hours, audiences cannot enjoy off-site information services during non-working hours [15]. In addition, due to the lack of cultural display means in the way of cultural exhibitions, many people usually walk around and ignore the rich cultural connotation of the exhibits during the viewing process, resulting in a huge waste of cultural resources. The cultural heritage, education, and communication value of cultural museums have not been properly promoted. Therefore, the construction of the digital science platform of the cultural center to strengthen the ability to protect cultural heritage, the cultural center's historical and cultural education, and communication function has been promoted, as well as its practical significance and far-reaching historical significance, as shown in Figure 1.

The implementation of edge computing relies on users to upload their computing tasks to the edge computing server, and this process can be subdivided into five steps: task discovery, offloading decision, uploading task, processing task, and returning results. The first step of task discovery refers to finding available edge computing servers, which are generally deployed on the base station side, and accessing the base station is considered as accessing the server. The offloading decision first includes whether to upload or not; this decision is mainly determined by the current communication computing resources; when the upload cost is lower than the local computing cost, the task chooses to upload. This is followed by the uploading scheme, i.e., whether to upload all or part of the task, which mainly depends on whether the system supports task splitting. When the task itself allows splitting, the user can choose to upload a certain percentage of files and calculate the remaining tasks locally. Task submission is the process of uploading data. In most scenarios, the terminal uploads the task to the base station via a wireless network, which itself involves no difference in technology from the ordinary wireless uplink. After the task is uploaded, the edge computing server opens a resource for the task to run through virtualization technology. After the task is calculated, the result is transmitted back to the terminal through the downlink channel. Diversity is a concept that is relevant to many fields of study, from ecology to information theory to economics, to name a few. The concept has received increasing attention in the information retrieval, network analysis, and artificial neural network communities. Although there are a growing number of applications using diversity measures in network structured data, there is no clear and comprehensive description of the different approaches to measuring diversity.

The quality of the wireless channel is greatly affected by the environment, and the uncertainty brought about by the reflection and scattering of radio signals is manifested by the fading phenomenon, and the electromagnetic wave fading is divided into large-scale fading and small-scale fading. Large-scale fading refers to the shadow effect and path loss of electromagnetic waves affected by the building terrain and other environmental effects [16]. Small-scale fading refers to the rapid fluctuation of electromagnetic waves received by the terminal antenna in a small range of movement. The electromagnetic wave transmission process will inevitably superimpose white noise, considering the channel loss of electromagnetic waves and superimposed noise:

(3) $$\begin{array}{c} \text{SNR}=\frac{hP}{\sigma^{2}}, \end{array}$$

where *Р* is the transmitting power at the transmitter, *h* is the gain of the signal throughout the transmission process, including path loss and antenna gain, and *σ*^2^ is the white noise power. The base station provides a fixed total uplink bandwidth, and the content to be optimized in the communication submodule is frequency allocation, there is no frequency multiplexing between terminals, and the uplink bandwidth can be continuously allocated in this scenario. The allocation scheme of communication bandwidth is decided by the search algorithm in the later section, and the allocation of communication resources is adjusted through iterations to finally make the system cost-optimal.

Considering only the remaining resources of the edge computing server, the following analysis is performed. The base station itself currently has the maximum allowable bandwidth *B* and the current remaining computing resources *F*. The end-user has a new task *J* to determine the offloading policy. For the pending task *J*, if offloading to the edge server is selected, the corresponding time overhead and end-user energy consumption are

(4) $$\begin{array}{c} T_{j}^{c}=T_{t}-T_{c},\\ E_{j}^{c}=T_{t}p_{j}^{2}, \end{array}$$

where *T*_*t*_ is the upload time for the calculation task and *T*_*c*_ is the calculation time for the task. Also available are

(5) $$\begin{array}{c} T_{t}=\frac{l_{j}^{2}}{r_{j}^{2}},\\ T_{c}=\frac{c_{j}^{2}}{f_{j}^{2}}. \end{array}$$

The edge computing server, in addition to new start-up devices, often has its old tasks unfinished, and previous studies have ignored the resources occupied by old tasks as unusable resources. Considering such a scenario, the terminal task itself latency tolerance is very high, but currently, the service area is in an idle period, the application for resources of the terminal is very little, in line with the principle of maximizing the revenue now, the system to allocate its full number of resources, and its expected completion time is less than the task regular latency requirements. In the process of the task execution, a new task request with very high latency requirements arrives, and the remaining computing resources of the system cannot meet its requirements at this time, and if the system cannot make new adjustments, the task can only be executed locally or fails. At this point, the old task is suspended due to the low latency gain and the empty resources are used to complete the new task, and the old task continues to execute after the new task is completed, making both tasks complete, increasing the system revenue or reducing the system cost. Applications for edge computing are initiated at the edge side, generating faster responses to network services and meeting the industry's essential needs for real-time services, application intelligence, security, and privacy protection. Edge computing sits between, or on top of, the physical entity and the industrial connection. Cloud computing, on the other hand, still has access to historical data from edge computing. Following the modeling process above, the scenario of joint optimization of the old and new tasks is modeled as follows.

**Figure 2:**
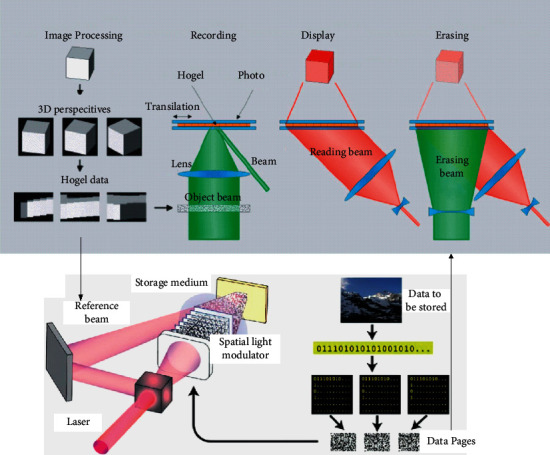
Principle of holographic projection.

The basic characteristics of the social network structure can be seen through the causality of system dynamics, but it does not reflect the cumulative nature of the variables. To better reflect these dynamics of variables, system state variables are defined in system dynamics to describe cumulative variables. Such state variables can reflect the operational state and outcome of the system and can also see the inventory or system state that is formed by accumulation in the system [17]. In system dynamics, the state variable changes with time, and the response can be expressed in the system as a rate or rate of change, so that this feedback loop in system dynamics operates in a flow-like process, as shown in Figure 2.

The 3D model allows users to better understand the objects from a three-dimensional perspective and better observe the detailed parts of the objects. It is difficult for users to understand the whole picture of the objects, rituals, or complex objects involved in intangible cultural heritage only using graphics, and the video can only make users passively accept it, but cannot form a way for users to actively explore the parts they are interested in or understand the details. This problem can be solved by displaying the 3D model, which allows users to view the details of the model in all directions. The user's operations for the 3D model are mainly zooming, rotating, and panning, with panning being a relatively low-frequency operation. The common interaction gestures in this area were single-finger sliding, two-finger stretching, two-finger dragging, etc. Therefore, the two-finger stretching is used to control the zooming in and out of the 3D model, the sliding after the single-finger press and hold is used to control the rotation of the model, and the two-finger pressing is used to achieve the panning of the model. The Internet promotes the realization of the value of excellent Chinese traditional culture. The value of traditional culture lies in its historical value, artistic value, intellectual value, and the values condensed in it. The Internet plays a catalytic role in the realization of the value of traditional culture.

In general, when designing digital nonfiction cultural products, the focus is on finding the right presentation for the information presented, which is the most important part of rich media interaction. This is the most important part of rich media interaction. The combination of appropriate presentation and user-friendly interaction gestures can bring the best user experience to users so that they can better feel the artistry and literature conveyed in the work.

### 3.2. Analysis of the Interactive Communication of Traditional Culture

The economic take-off, the progress of social development, and the deepening of globalization and modernization, coupled with the rise of the Internet era and other elements make today's social development, present a diverse and complex situation. Cultural elements are the concentrated representatives of traditional culture, the cultural treasures precipitated by China's historical development, and the collective identity reached by the people in their subtle habits. However, the source of the existence and development of cultural elements still comes from the soil built on the political level, economic level, and lifestyle of ancient Chinese society and is a source of constant ideological and cultural watering obtained in the longitudinal history. Today's society has long since entered a modern civilization, an era of true speed, and convenience [18]. The ancient culture for modern society and modern people, like antiques on display in museums, is only the imprint left by history. Traditional culture is sweeping in the current surging social wave of rampant development. In the industrialized era, the contemporary social civilization of market economy and pan entertainment is being eroded and dissipated. Cultural elements as historical culture and symbols are gradually fading out. The cultural heritage of contemporary society is facing great difficulties.

**Figure 3:**
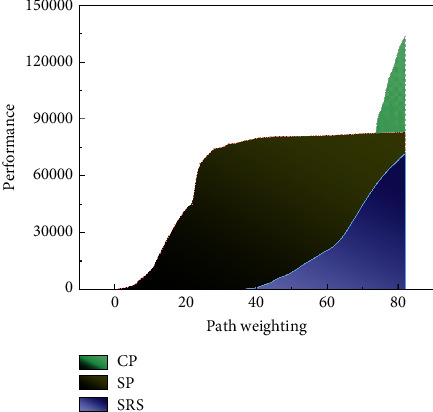
Performance comparison of fused meta-paths with different path weights.

In addition, the leapfrog development of mobile Internet has enabled people to enter this cyberspace utilizing mobile terminals, which are free from the limitations of traditional computers and enable information not only to penetrate more audience groups of different classes vertically but also to have wider geographical and country-specific coverage horizontally. In this kind of mobile network space, people can interact, share, and communicate without time and space limitations, and it is easier for them to develop cultural and emotional identities. On the other hand, the quality of mobile Internet makes people break through the barrier of time and space and brings a higher sense of community identities such as nation and country, and this sense of identity is expanded in the broadest scope in the mobile network space. It can be said that the real-time experience sharing and information exchange communication methods brought by the mobile Internet precisely transcend the boundaries of nations and nationalities and continue to influence participants' national emotional identity, identity orientation, and the formation of national conceptual patterns in a global span. It is in breaking through the barriers of time and space and building an environment of all-time connectivity that the mobile Internet influences changes in social form while creating the best conditions and environment for the digital art dissemination of cultural elements, as shown in Figure 3.

In the fusion meta-path, the path weights represent the contribution of different single meta-paths to the fusion meta-path, and the choice of their values has an impact on the clustering performance. For the fusion meta-path SRS + SCS, the sum of its path weights is 1. In this paper, the path weights of every single meta-path are varied according to a gradient of 0.1, so that different evaluation results are obtained. The evaluation results are shown in Figure 3, where *w*_*p*_1__, the path weights of the meta-path SRS, and *w*_*p*_2__, the path weights of the meta-path SCS, are represented.

Different path weights have different effects on the clustering results, and the best clustering results are obtained when *w*_*p*_1__ and *w*_*p*_2__ are 0.7 and 0.3, respectively, which also indicates that better clustering results can be obtained when the meta-path SRS contributes more to the fused meta-paths. As the path weight of a single meta-path approaches 1, the fused meta-paths are closer to the case of using only single meta-paths, and the effectiveness of the algorithm starts to decrease.

**Figure 4:**
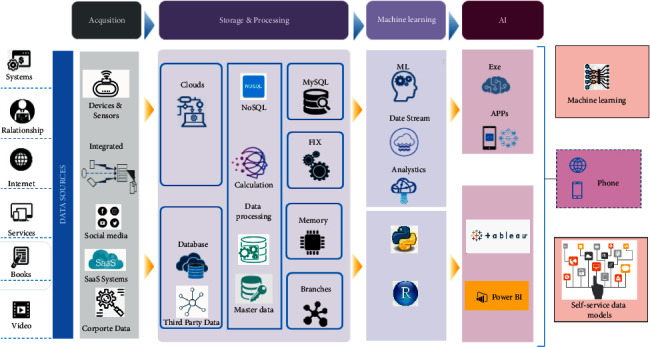
Interactive communication model of traditional culture.

The human brain has certain characteristics of the cognition of visual information; the computer through the analysis and calculation of this characteristic can make people form a picture close to the real sensory perception; although the visual graphics that people look at are based on the digital system virtual display, through data analysis, it will give people a super shocking visual journey through [19]. This virtualized display is no longer just a combination of points, lines, and surfaces, but the use of digital technology, through audio-visual technology, graphic display technology, dynamic simulation technology, in the display space to create a scene consistent with the real environment, so that the audience completely mobilizes all the sensory systems of the body, so that the space and people merge in the real and unrealistic visual space as shown in Figure 4.

Although the virtual space formed by the phantom image or projected by the projector cannot be extended to the space of the real exhibition hall or exhibits, it can quickly establish the visual space to expand the psychology and create a strange atmosphere and enough spatial hierarchy to create a dreamy and profound atmosphere. The “vertical surface” formed by the virtual phantom is a visually impactful interface that people can visit in the virtual interface, yet the visual and spatial aspects do exist. The audience in this dynamic virtual objective time and space is real and unreal, fantastic, and interesting.

Upon entering the music and dance scene, the camera will first take a general view from top to bottom and finally zoom in to the middle of the screen. The scene presents the layout of people and instruments in the music and dance scene in front of Dacheng Hall, including the placement of dance students, music students, song students, and the instruments used [20]. User can control the zoom and permission of the page by stretching and contracting two fingers on the page and can drag the page by pressing and holding the page slide finger. Click one of the instruments or people, and user will jump to the corresponding detailed introduction page The details of the musicians and dancers on the stage of Dacheng Hall during the three dedications are displayed comprehensively.

## 4. Analysis of Results

### 4.1. The Internet Convergence Digital Media Performance Results

After the fixed parameters initially reflect the benefits brought by the old and new tasks of WOA optimization, to further observe the impact of different factors on the system cost of this system from a multi-dimensional perspective, the control variable method can be used; i.e., except for the selected variable parameters, the remaining parameters are still designed according to the parameters in the parameter table. Many factors are affecting the edge computing system cost, and factors affecting both communication and computation can affect the final system cost [21]. The impact of changing the number of computing resources, communication resources, and terminals, i.e., the number of tasks, on the system cost, is compared and analyzed separately. Computational resources are selected from the total CPU processing capacity of the edge server, which is the most critical resource for MEC to provide computing services to the outside world, and communication resources are selected from the total bandwidth provided by the base station, which is the core resource in wireless communication. These two parameters are the key indicators that affect user experience in the MEC system, so they are varied to observe the curve changes.

**Figure 5:**
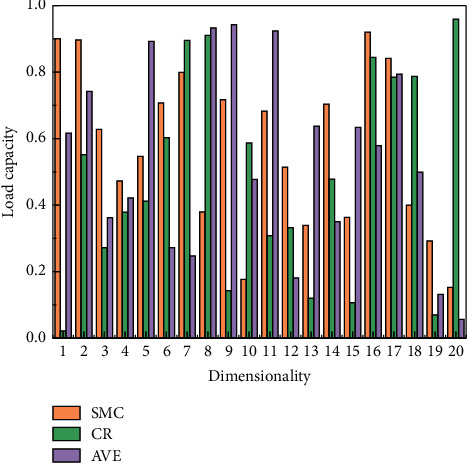
Standardized load volume report.

The validating factor analysis is used to comprehensively test whether the theoretical model is consistent with the empirical data and whether the theoretical model has applicability and truthfulness. The SPSS22.0 and AMOS17.0 software were used to perform validating factor analysis in conjunction with structural equation modeling. This study uses the method of releasing one parameter at a time, modifying its fixed parameter values to free parameters, and re-estimating the model. After several corrections to the theoretical model, the model test results were finally accepted with the discriminant validity, and the standardization coefficients are shown in Figure 5.

**Figure 6:**
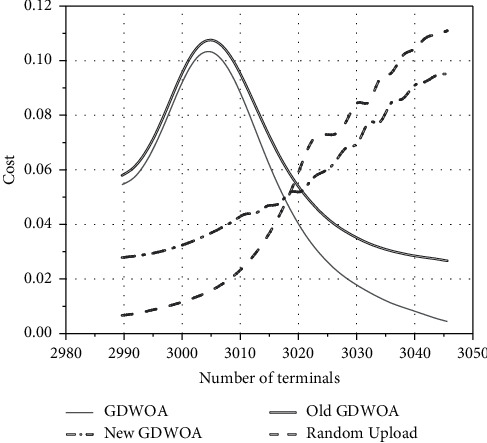
Number of terminals-system cost relationship.

Figure 6 shows that the whale optimization algorithm can bring significant gains, especially when jointly optimizing old and new tasks can always bring partial gains, and the gap between the two curves of whether to optimize old resources gradually shrinks as the computational resources increase. Because the bottleneck brought by the computational resources is gradually broken when the computational resources are gradually increased, the system does not need to sacrifice the experience of running tasks for some of the gains, and the gains brought by jointly optimizing old and new tasks are no longer significant. The increase in computing resources greatly reduces the system cost of the all-upload scheme, which is the most demanding one in terms of resources for edge computing facilities. When computing resources are small, the scheme is even inferior to local computing because it ignores local computing resources and all tasks are crowded to the limited edge computing servers. The all-local is the worst solution in most cases, and it completely forgoes the huge potential benefit of hardware cost for edge computing [22]. The random upload scheme simply considers the limited edge computing resources and performs better than all uploads and all local.

As seen in the figure, when the offload decision is determined, i.e., all-local, all-upload, and random-upload scenarios, the energy cost is constant, which is because the upload energy consumption and local energy consumption are not affected by the MEC computing resources when the offload policy is determined. The change in MEC computing resources affects the processing time of the offloaded task in the server, and as the MEC computing resources increase, the sum of time is on a decreasing trend. The whale optimization algorithm has a clear advantage that its strategy of jointly optimizing the old and new tasks always results in the optimal one. The upload strategy has the worst effect when the computational resources are small, and as the computational resources are gradually relaxed, the time of the all-upload strategy gradually decreases and becomes the second-worst strategy.

As can be seen in Figure 6, the whale algorithm jointly optimizing the old and new tasks is always the optimal strategy, and the advantage of this strategy is not obvious when the number of terminals is small. Compared with optimizing only the new tasks, the two solutions almost overlap when the number of terminals is relatively small, but a closer look at the curve shows that this strategy is still the optimal solution. As the number of terminals increases, increased tasks initiate resource requests to the edge computing server, and the advantages of jointly optimizing old and new tasks are gradually demonstrated, and the difference between the two curves gradually increases. When the number of terminals is not large, all tasks are computed locally, which is the worst solution and completely wastes the abundant resources provided in the system. The system cost of local computation increases almost linearly as the number of terminals rises, and as the number of terminals rises, the local resources wasted by the all-upload scheme gradually increase, and its system cost eventually exceeds the all-local curve to become the worst scheme. And when the number of terminals increases, the all-task upload equal share resource scheme becomes the worst scheme because of the wasted local computing resources in this scheme. Like the results in Figure 6, the random uploading strategy performs moderately well, and its cost is the best among the three nonoptimized strategies, but the difference with the optimized curve is obvious. This inspires us to consider using systems such as tokens to randomly triage the endpoints requesting resources in our optimization-free engineering practice, which can also yield better results.

### 4.2. Results of the Interactive Transmission of Traditional Culture

**Figure 7:**
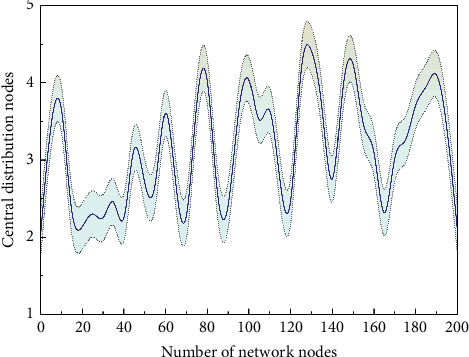
Distribution of intermediate centrality of network nodes.

From the comparison between minds and social networking sites, the users with higher intermediate centrality of the blockchain platform are less related to whether they are authenticated or not, but closely related to the value of the virtual currency they receive, while the users with higher intermediate centrality of social networking sites are closely related to whether they are authenticated or not and whether they are authoritative media. It indicates that the dissemination of blockchain public opinion information, because it is based on the advantages of blockchain technology, reduces the trust among users and concerns about the security and credibility of information and focuses more on the quality of information. Thus, the blockchain opinion platform with higher intermediate centrality users is the bridge of opinion dissemination in the real sense and is relying on the quality of opinion information to achieve an irreplaceable position in the network. From the comparison of the distribution curves of intermediate centrality, the distribution of intermediate centrality of blockchain opinion network nodes is more regular and shows obvious tail-dumping characteristics, as shown in Figure 7.

The application of artificial intelligence technology in the visual design of the exhibition hall should be based on the intelligent attributes of artificial intelligence technology as the support and give the exhibitors the most personalized intelligent visual experience through intelligent visual expression. The uniqueness of the visual language should be integrated with intelligent technology, and intelligent technology should be reflected through the visual language. On the one hand, it is necessary to ensure the distinctive and unique visual expression style of the exhibition hall, and on the other hand, it is necessary to consider the intelligent visual needs of the audience. Any innovative expression in concept, form, and function of the exhibition hall should be based on how to better meet the practical needs of the audience, and on this premise, the creative expression should be made according to the space layout of the exhibition hall and the characteristics of the exhibits themselves. The application and integration of artificial intelligence technology in the visual design of the pavilion make the visual language of the pavilion design transform in real-time according to the changes of the audience and the real environment, to give the audience the most suitable creative visual language expression.

**Figure 8:**
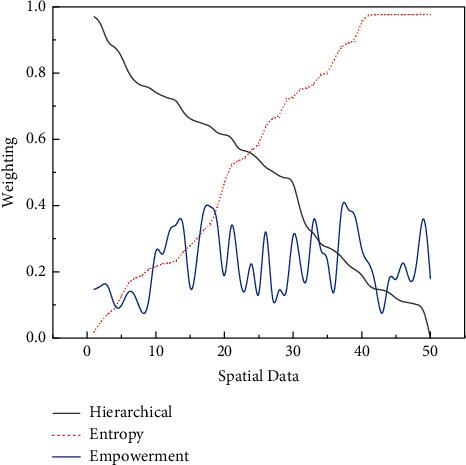
Optimal assignment to calculate weights.

As mentioned earlier, appropriate breakout spaces can be embodied in modern showrooms in a flexible way to make the magic of AI technology more enjoyable for users. After visiting the exhibits, the user experience will undoubtedly be enhanced if they can further utilize the communication space in the showroom for better relaxation and learning. Specifically, the visual design of the rest space in the exhibition hall is to use AI technology to transform the data related to the rest area (such as spatial openness, spatial environment, furnishings, and functional area relationship) into a 3D virtual model, so that users can experience the rest space more efficiently, as shown in Figure 8.

The highest efficiency of information acquisition is the paper-based graphic material, probably because the paper-based graphic material is specially organized and presented, from which the information needed in the test questionnaire can be quickly found, which makes users feel that this method is the most efficient way to obtain information. In the overall experimental process, it was found that one of the obvious problems was that the rich media interaction method improved the interestingness of the work, but the improved interestingness inevitably reduced the popularization of science, which affected the efficiency of information acquisition by users. Therefore, how to improve the fun while reducing the loss of science, so that users can obtain information and knowledge more quickly and accurately, will be a direction to focus on in the next exploration.

## 5. Conclusion

The digital media is a young and dynamic player in the public culture service. It is not only a form of digital business but also a mobile service and full coverage of cultural hall position service. Cultural centers can break the traditional concept of cultural services, provide the public with a variety of rich cultural products that meet their expectations, expand a wider space for public cultural services, and meet the actual cultural needs of people to enjoy. In an environment of cross-disciplinary integration of technology and art, digital culture centers provide more advanced, extensive, convenient, and equitable public cultural services to the public with their new service models and colorful cultural products. In this virtual exhibition hall, the cost of services is effectively controlled, various effective resources are utilized, the audience is effectively expanded, and the government's cultural benefit policy is widely realized. Letting digital culture enter thousands of households will eventually become a reality. In today's world where communication power is productivity and creativity, it is to establish an international communication system that is balanced and matched with its political status, economic strength, and international influence to be able to fully present cultural civilization, tell a good story, and present an in-depth, three-dimensional national image to the world. The new stage of Internet development has created opportunities for the realization of this goal, and digital art communication of cultural elements representing the cultural lineage is one of the paths, and the best path to follow the current trend of the times.

## Data Availability

The data used to support the findings of this study are available from the author upon request.
